# Antibacterial Activities of Pyrenylated Coumarins from the Roots of *Prangos hulusii*

**DOI:** 10.3390/molecules22071098

**Published:** 2017-07-01

**Authors:** Nur Tan, Seçil Yazıcı-Tütüniş, Merve Bilgin, Emir Tan, Mahmut Miski

**Affiliations:** 1 Department of Pharmacognosy, Faculty of Pharmacy, Istanbul University, Istanbul 34116, Turkey; zecilyazici@hotmail.com; 2 Department of Pharmaceutical Microbiology, Faculty of Pharmacy, Istanbul Yeni Yuzyil University, Istanbul 34110, Turkey; merve.bilgin@yeniyuzyil.edu.tr (M.B.); emir.tan@yeniyuzyil.edu.tr (E.T.)

**Keywords:** *Prangos hulusii*, pyrenylated coumarins, antibacterial activity

## Abstract

The dichloromethane extract of the roots of *Prangos hulusii*, a recently described endemic species from Turkey, has yielded nine known and one new prenylated coumarins. The structures were elucidated by spectroscopic methods and direct comparison with the reference compounds where available. The root extract and its prenylated coumarins exhibit antimicrobial activity against nine standard and six clinically isolated strains at a concentration between 5 and 125 µg/mL. In particular, the new coumarin, 4′-senecioiloxyosthol (**1**), displayed 5 µg/mL MIC (Minimum Inhibitory Concentration) value against *Bacillus subtilis* ATCC 9372, murraol (**4**) and auraptenol (**5**) showed 63 µg/mL MIC value against *Klebsiella pneumoniae* ATCC 4352 and *Bacillus subtilis* ATCC 9372, and isoimperatorin (**9**) exhibited 16 µg/mL MIC value.

## 1. Introduction

*Prangos* is an important genus of Apiaceae family, with 43 species known worldwide [1]. There are 17 species of *Prangos* in Turkey; nine of them are endemic [2]. Members of this genus have carminative, laxative, stomachic, stimulant, emmenagogue, antienflammatuar, antimicrobial, and antidiabetic properties, and are used for the treatment of burns, hemorrhoids, and wounds [3,4,5,6]. Many coumarin, alkaloid, flavonoid, and terpenoid derivatives were isolated from the roots, aerial parts, and fruits of *Prangos* species [7,8,9,10]. *Prangos hulusii* (S. G. Şenol, H. Yıldırım & Ö. Seçmen) is a newly identified endemic species from Flora of Turkey [11]. Preliminary biological activity studies on the extracts of the roots of *P. hulusii* showed the presence of antimicrobial and cytotoxic activities [12]. 

**Figure 1:**
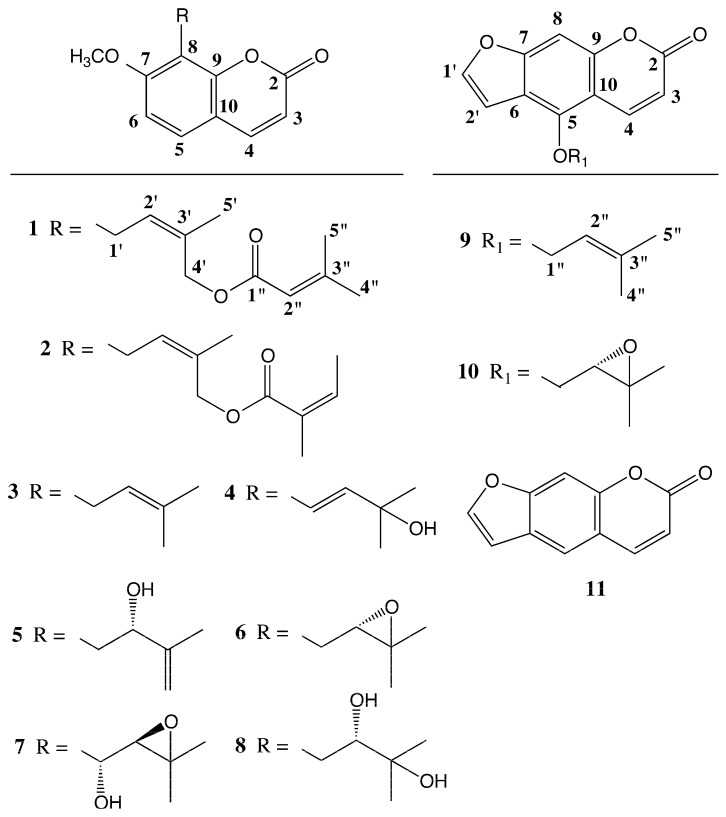
Structures of prenylated coumarins **1**–**10**.

The dichloromethane extract of the roots of *P. hulusii* was subjected to a series of chromatographic separations to yield a new coumarin, 4′-senecioiloxyosthol (**1**), along with nine known coumarins; osthol (**3**) [13], murraol (**4**) [14], auraptenol (**5**) [15], meranzin (**6**) [16], hydroxyosthol-epoxide (**7**) [17], meranzin hydrate (**8**) [16], isoimperatorin (**9**) [18], oxypeucedanin (**10**) [18], psoralen (**11**) [19], and two phytosterols; stigmasterol and β-sitosterol [20]. Structures of the isolated compounds (Figure 1) were elucidated using spectroscopic techniques and chemical transformations as well as by direct comparison with the reference standards where available. Antimicrobial activities of the dichloromethane extract of the roots of *P. hulusii* and prenylated coumarins isolated from this extract were investigated against the standard and clinically isolated 15 bacterial strains. 

## 2. Results and Discussion

**Figure 2:**
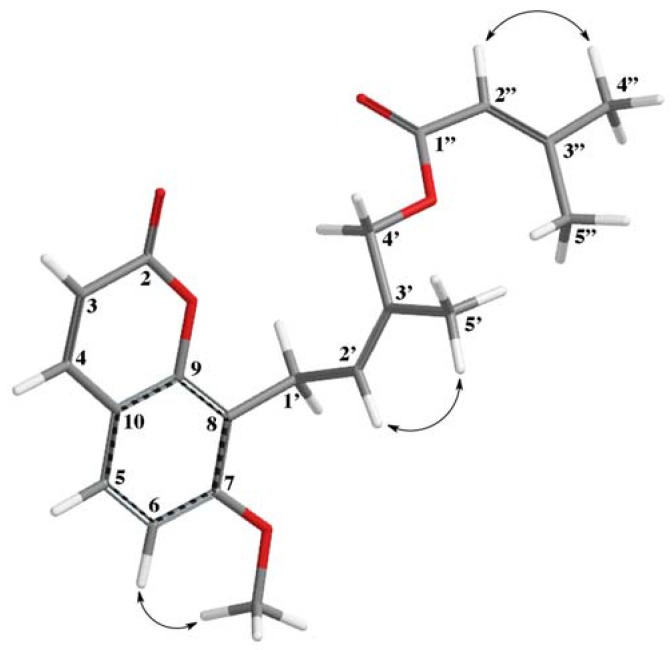
Interactions observed in the ROESY spectrum of 4′-Senecioiloxyosthol.

4′-Senecioiloxyosthol (**1**) was obtained as a colorless gum. The HRESIMS spectrum of **1** suggests a molecular formula of C_20_H_22_O_5_ with 10 degrees of unsaturation based on the [M + H]^+^ molecular peak at *m*/*z* 343.1544 (calcd *m*/*z* 343.1545). The ^1^H-NMR spectrum (Table 1) of **1** was very similar to that of osthol [13,16], with the exception of a missing vinylic methyl group signal of the prenyl side chain of osthol. Instead of two vinylic methyl signals, the ^1^H-NMR spectrum of **1** displayed a vinylic methyl group signal at δ_H_ 1.72 (3H) and a methylene singlet at δ_H_ 4.87 (2H), indicating that the second vinylic methyl group of osthol side chain was replaced with an acyloxy bearing methylene group. Furthermore, the typical vinylic narrow quintet proton signal observed at δ_H_ 5.72 (*J* = 1.3 Hz) along with the two vinylic methyl group doublets at δ_H_ 2.19 and 1.90 (each 3H, *J* = 1.3 Hz) suggest the presence of a senecioil group as the acyl group. The 2D-ROESY spectrum of **1** exhibited interactions between C-5′ methyl group protons and H-2′ proton of the prenylated side chain of osthol (Figure 2) as well as displayed interactions between H-6 and the methoxy group protons at C-7, and H-2″ proton and H-4″ methyl protons of the senecioiloxy acyl group, which clearly confirms the presence of a senecioiloxy acyl group at the C-4′ methyl group of osthol in **1**. Furthermore, ^13^C-NMR (Table 1), 2D COSY, UV and IR spectroscopic data (see experimental section and supplemental data) of **1** corroborated the structure as 4′-senecioiloxyosthol. Previously, 4′-angeloiloxy derivative of osthol (**2**) (i.e., macrocarpin) was reported from *Lomatium macrocarpum* (Hook. & Arn.) C. & R., another Apiaceaen plant [21]. The ^1^H-NMR spectroscopic data reported for macrocarpin (**2**) were similar to that of **1** with the exception of the presence of angeloiloxy acyl group signals [i.e., δ_H_ 6.04 (1H, br t), 1.97 (3H) and 1.88 (3H)] instead of a senecioiloxy acyl group signals. 

The antimicrobial activity of extracts and isolated coumarins of *Prangos hulusii* was evaluated against Gram-positive and Gram-negative nine reference standards and six clinically isolated microorganism strains. The results of minimum inhibition concentration (MIC, in µg/mL) values are summarized in Table 2. The best antimicrobial activity was observed against *Escherichia coli* with the dichlormethane (DCM) extract (i.e., MIC at 156 µg/mL), followed by the petroleum ether (PE) and methanol (MeOH) extracts (i.e., each MIC at 313 µg/mL). All three extracts showed good activity against *Enterococcus faecalis* (MIC at 313 µg/mL). Similar activities were detected with the DCM extract against *Proteus mirabilis*, with the PE extract against *Staphylococcus aureus* and with the MeOH extract against *Klebsiella pneumoniae* ATCC 4352*.* No activity was observed with all of the tested extracts against clinical isolates *K. pneumoniae*, *Acinetobacter baumannii*, and *E. coli*, and only a weak activity was detected against other reference and clinical isolate bacteria. 

The new coumarin, 4′-senecioiloxyosthol (**1**), showed the best activity against *Bacillus subtilis* (Table 2) (MIC at 5 µg/mL), whereas the structurally related osthol (**3**) and isoimperatorin (**9**) displayed very good activity against clinical isolate Methicillin-resistant *Staphylococcus aureus* (MRSA) (MIC at 16 µg/mL), similar to the reference antibiotic Cefotaxime (CEF) (see Table 2). In contrast, auraptenol (**5**) and murraol (**4**) exhibited good activity against *K. pneumoniae* ATCC 4352 and *Bacillus subtilis* ATCC 9372 (MIC at 63 µg/mL) and poor activity against Methicillin Resistant Coagulase-Negative *Staphylococci* (MRCNS) (MIC at 125 µg/mL). Furthermore, auraptenol (**5**) displayed a good activity against *Staphylococcus epidermidis* ATCC 12228 and MRCNS (MICs at 63 and 125 µg/mL, respectively), osthol (**3**) against *B. subtilis* ATCC 9372, *S. aureus* ATCC 25923, *K. pneumoniae* ATCC 4352 and Methicillin-sensitive *Staphylococcus aureus (*MSSA) (all MICs at 125 µg/mL). 

The antimycobacterial activity of prenylated coumarins and prenylated furanocoumarins [22,23] as well as the antimicrobial activity of furanocoumarins and prenylated furanocoumarins were reported previously [24]. In the latter publication, xanthotoxin (8-methoxyfuranocoumarin) was described as the most potent compound against *B. subtilis* ATCC 6633 strain with an MIC value at 30 µg/mL, whereas the new prenylated coumarin 4′-senecioiloxyosthol (**1**) was 6-fold more active against *B. subtilis* ATCC 9372 than that of xanthotoxin, with an MIC value at 5 µg/mL.

## 3. Materials and Methods

### 3.1. General Experimental Procedures 

UV spectra were recorded on a UV-1700 PharmaSpec Shimadzu spectrophotometer (Shimadzu Corp., Kyoto, Japan) in MeOH. IR spectra were measured with a PerkinElmer Spectrum 2000 FT-IR spectrometer (PerkinElmer Corp., Waltham, MA, USA). NMR experiments were conducted on a Varian Mercury FT-NMR 400 MHz spectrometer (Agilent Corp., Santa Clara, CA, USA) using tetramethylsilane (TMS) as an internal standard. High Resolution Electrospray Ionization Mass Spectra (HRESIMS) and Electrospray Ionization Mass Spectra (ESIMS) were determined on Waters SYNAPT G1 mass spectrometer (Waters Corp., Milford, MA, USA). 

### 3.2. Plant Material 

The roots of *Prangos hulusii* were collected from Ödemiş by Hulusi Kütük, İzmir on March 2012, in Turkey. The plant was identified by Professor Emine Akalin Uruşak and a voucher specimen was deposited in the Herbarium of Istanbul University, Faculty of Pharmacy (ISTE 99676). 

### 3.3. Isolation of Compounds

The dried and coarsely powdered roots (835 g) were exhaustively extracted with PE, DCM and MeOH, sequentially, using a Soxhlet Apparatus. The solvents were evaporated under reduced pressure in a rotary evaporator. The dichloromethane extract (55.6 g) was dissolved in acetone (2000 mL), and kept in a refrigerator overnight. Following the removal of precipitated hydrocarbon mixtures by filtration, the solvent was removed in vacuo to yield 45.8 g viscous oil. A portion of the defatted extract (5 g) was chromatographed on a Sephadex LH-20 (5 cm × 60 cm) packed in hexane-dichloromethane-methanol (7:4.5:0.5) and Preparative Thin Layer Chromatography (prep. TLC) (1-2 mm thickness, silica gel developed with cyclohexane-EtOAc mixtures, 4:1, 3:2, 1:1) was used for the final purification of compounds. 4′-senecioiloxyosthol (**1**) (4.7 mg), osthol (**3**) (39 mg), murraol (**4**) (5 mg), auraptenol (**5**) (8.2 mg), meranzin (**6**) (7 mg), hydroxyosthol-epoxide (**7**) (4 mg), meranzin hydrate (**8**) (12 mg), isoimperatorin (**9**) (23 mg), oxypeucedanin (**10**) (60 mg), psoralen (**11**) (7 mg), stigmasterol (3 mg), and β-sitosterol (2.8 mg) were isolated. Furthermore, meranzin (**6**) (11.3 mg), meranzin hydrate (**8**) (10.2 mg), and auraptenol (**5**) (7.6 mg) were prepared from osthol (**3**) semi-synthetically [16] as a reference material.

### 3.4. 4′-Senecioiloxyosthol (**1**)

IR (KBr) ν_max_: 2970, 2915, 2842, 1732, 1717, 1651, 1608, 1281, 1251, 1226, 1145, 1118, 1090 and 832 cm^−1^. UV (MeOH) λ_max_ (log ε) 321 (4.06), 258(sh) (3.82), 247 (3.26) and 224(sh) (3.81) nm. For ^1^H (CDCl_3_, 400 MHz) and ^13^C-NMR (CDCl_3_, 100 MHz) spectroscopic data, see Table 1; HRESIMS *m*/*z*: 343.1544 (calcd for C_20_H_23_O_5_ 343.1545).

### 3.5. Antimicrobial Activity 

The antimicrobial activity of the extracts and isolated coumarins of *Prangos hulusii* was evaluated against nine reference standard microorganisms, both Gram-positive and Gram-negative; *S. aureus* ATCC 25923, *S. epidermidis* ATCC 12228, *E. faecalis* ATCC 29212, *Pseudomonas aeruginosa* ATCC 27853, *E. coli* ATCC 10799, *K. pneumoniae* ATCC 4352, *Salmonella choleraesuis* ATCC 14028, *P. mirabilis* ATCC 7002, *B. subtilis* ATCC 9372*,* and six clinical isolates; (MSSA), (MRSA), (MRCNS), *K. pneumoniae*, *A. baumannii*, *E. coli*, by using a standard microbroth dilution method modified with rezasurin [25,26]. The experiments were performed with two replications and the results were expressed as average values.

### 3.6. Determination of Antibacterial Activity

The MIC values of extracts and isolated compounds were determined using microbroth dilution method in 96-well microtitre plates. The bacterial cultures were prepared from overnight cultures on Tryptic Soy Agar (TSA) at 37 °C for 24 h by diluting in Mueller Hinton Broth (MHB) from approx. 10^8^ CFU/mL to 2 × 10^6^ CFU/mL. Then, 50 μL Mueller Hinton Broth (MHB) was added to the wells starting from the first well and continuing up to the twelfth. The extracts and isolated compounds were prepared 1/10 (*v*/*v*) in DMSO and 50 μg/mL of these were added to the first wells. Two-fold serial dilutions were made, achieving a final concentration ranging from 5000 to 10 μg/mL. The positive controls for Ciprofloxacin (CPR), Tetracycline (TTR), Cefotaxime (CEF), and Oxacillin (OXA) were determined with the final concentrations from 64 to 0.1 µg/mL. In addition, an extra row of DMSO was used as a vehicle control to determine its possible inhibitory activity. Finally, 25 μL of bacterial suspensions and % 0.001 resazurin solution were added to each well. 

After incubating the bacteria at 37 °C for 24 h, the microtitre plates were examined visually for microbial growth which appeared as pink, colored by resazurin dye. In each row, the well containing the least concentration that showed no visible growth was considered the MIC. The bacterial samples were inoculated on TSA plates and incubated at 37 °C for 24 h.

## 4. Conclusions

Investigation of the dichloromethane extract of the roots of *P. hulusii* yielded several pyrenylated coumarins and furanocoumarins with antimicrobial activities. *Prangos* species frequently used for the treatment of burns and wounds in traditional folk medicine [3,4,5,6], perhaps the presence of pyrenylated coumarins with antimicrobial activity may play an important role for the aforementioned folkloric use of *Prangos* species. 

## Figures and Tables

**Table 1:** ^1^H-NMR and ^13^C-NMR data of Compound **1**.

Positions	Δ_H_ (*J* in Hz)	Δ_C_, Type
2		161.14, C
3	6.23 d (9.8), 1H	116.08, CH
4	7.60 d (9.8), 1H	143.65, CH
5	7.30 d (8.4), 1H	126.46, CH
6	6.82 d (8.4), 1H	107.3, CH
7		160.09, C
8		116.66, C
9		152.86, C
10		112.92, C
-OCH_3_	3.89 s, 3H	56.01, CH_3_
1′	3.62 d (7.9), 2H	21.45, CH_2_
2′	5.50 br t (7.9), 1H	113.04, CH
3′		131.24, C
4′	4.87 s, 2H	62.40, CH_2_
5′	1.72 br s, 3H	21.61, CH_3_
1″		166.8, C
2″	5.72 quint (1.3), 1H	126.51, CH
3″		156.41, C
4″	1.90 br d (1.3), 3H	27.39, CH_3_
5″	2.17 br d (1.3), 3H	20.2, CH_3_

**Table 2:** Antimicrobial activity of the prenylated coumarins and root extracts of *P. hulusii*.

Test Strains	Prenylated Coumarins (μg/mL) **								Extracts (μg/mL) ***			References ****			
	1 *	3	4	5	6	8	9	10	DCM	PE	MeOH	TTR	OXA	CEF	CPR
	Minimum Inhibitory Concentration (MIC) μg/mL														
*S. epidermidis* ATCC 12228	625	>125	>125	63	>125	125	>125	125	625	313	625	64	0.5	4	0.5
*S. aureus* ATCC 25923	625	125	>250	>250	>250	250	>250	250	1250	625	1250	0.5	0.5	2	1
*E. faecalis* ATCC 29212	313	>250	>250	>250	>250	250	>250	250	313	313	313	16	8	2	1
*K. pneumoniae* ATCC 4352	313	125	63	63	125	250	125	250	625	625	313	8	4	2	0.5
*B. subtilis* ATCC 9372	5	125	63	63	>250	250	>250	125	1250	1250	1250	0.3	0.1	0.5	0.5
*E. coli* ATCC 10799	313	>125	>125	>125	>125	250	>125	250	156	313	313	2	32	1	0.3
*P. aeruginosa* ATCC 27853	156	>125	>125	>125	>125	250	>125	125	313	313	625	32	>64	16	0.5
*S. choleraesuis* ATCC 14028	313	>125	>125	>125	>125	125	>125	125	625	625	1250	4	>64	0.3	0.3
*P. mirabilis* ATCC 7002	313	>125	>125	>125	>125	250	>125	250	313	625	625	64	32	1	1
Methicillin-sensitive *Staphylococcus aureus* (MSSA)	625	125	>250	>250	>250	250	>250	250	1250	1250	2500	64	64	2	0.1
Methicillin-resistant *Staphylococcus aureus* (MRSA)	625	16	>250	>250	>250	250	16	125	1250	1250	2500	8	8	16	0.5
Methicillin Resistant Coagulase-Negative *Staphylococci* (MRCNS)	313	>250	125	125	125	125	>250	125	1250	1250	1250	8	8	16	4
*K. pneumoniae*	313	>125	>125	>125	>125	250	>125	250	2500	2500	2500	2	>64	>64	2
*A. baumannii*	n.t.	>125	>125	>125	>125	125	>125	125	2500	1250	2500	n.t.	n.t.	n.t.	>16
*E. coli*	156	>125	>125	>125	>125	250	>125	250	2500	2500	2500	>64	>64	>64	0.5

Starting concentrations: * 1250 μg/mL; ** 250 μg/mL; *** 5000 μg/mL; **** 64 μg/mL. TTR = Tetracycline, OXA = Oxacilline, CEF = Cefotaxime, CPR = Ciprofloxacin. n.t.: not tested PE: petroleum ether; DCM: dichlormethane; MeOH: methanol.

## Supplementary Materials

Supplementary materials containing spectroscopic data of the new coumarin are available online.
