# Supplementary material for: Antibacterial Activities of Pyrenylated Coumarins from the Roots of Prangos hulusii
Source: Molecules. 2017 Jul 1;22(7):1098. doi: 10.3390/molecules22071098 (PMC6152357; doi:10.3390/molecules22071098)
Supplement: Supplementary file 1 [file molecules-22-01098-s001.pdf]

SUPPLEMENTARY INFORMATION

Antibacterial Activity of Pyrenylated Coumarins from the Roots of *Prangos hulusii*

Nur Tan [1,*], Seçil Yazıcı-Tütüniş [1], Merve Bilgin [2], Emir Tan [2] and Mahmut Miski [1,*]

[1] Department of Pharmacognosy, Faculty of Pharmacy, Istanbul University, Istanbul 34116, Turkey; zecilyazici@hotmail.com

[2] Department of Pharmaceutical Microbiology, Faculty of Pharmacy, Istanbul Yeni Yuzyil University, Istanbul 34110, Turkey; merve.bilgin@yeniyuzyil.edu.tr (M.B.); emir.tan@yeniyuzyil.edu.tr (E.T.)

* Correspondences: nurtan@istanbul.edu.tr (N.T.); mahmut.miski@gmail.com (M.M.); Tel.: +90-530-491-6629 (N.T.); +90-545-550-4455 (M.M.)

Contents:

Figure S1. UV spectrum (in MeOH) of 4’-Senecioiloxyosthol (**1**).

Figure S2. IR spectrum (in KBr) of 4’-Senecioiloxyosthol (**1**).

Figure S3. ^{1}H-NMR spectrum (400 MHz, in $CDCl_3$) of 4’-Senecioiloxyosthol (**1**).

Figure S4. ^{13}C-NMR spectrum (100 MHz, in CDCl3) of 4’-Senecioiloxyosthol (**1**).

Figure S5. ^{1}H -^{1}H COSY spectrum of 4’-Senecioiloxyosthol (**1**).

Figure S6. ROESY spectrum of 4’-Senecioiloxyosthol (**1**).

Figure S7. HRESIMS spectrum of 4’-Senecioiloxyosthol (**1**).

Figure S8. ESIMS spectrum of 4’-Senecioiloxyosthol (**1**).

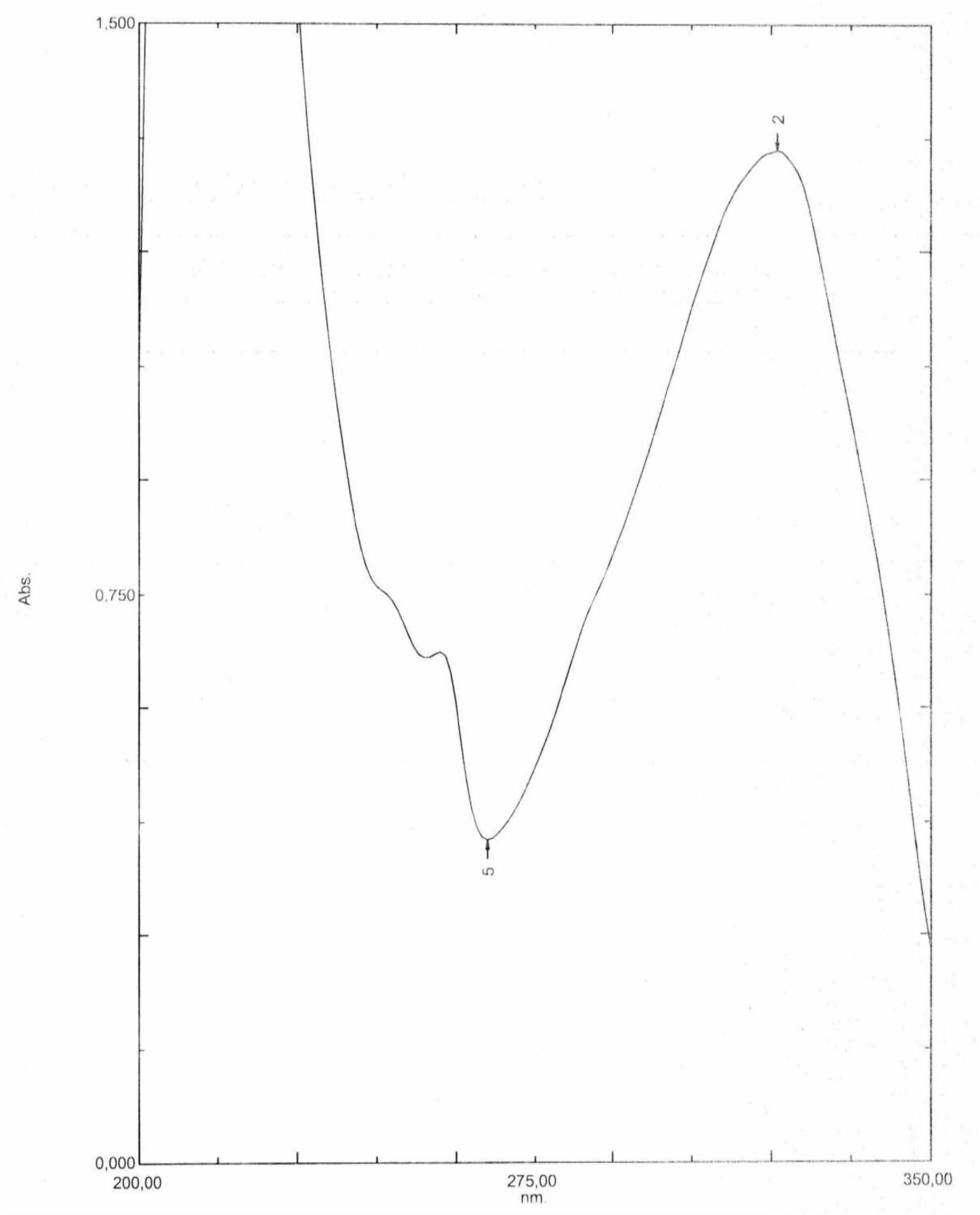

Wavelength (nm)	Absorbance	log ε
321	1.333	4,06
288 (sh)	0.768	3,82
257	0.673	3,76
247 (sh)	0.749	3,81

Figure S1. UV spectrum (in MeOH) of 4’-Senecioiloxyosthol (**1**).

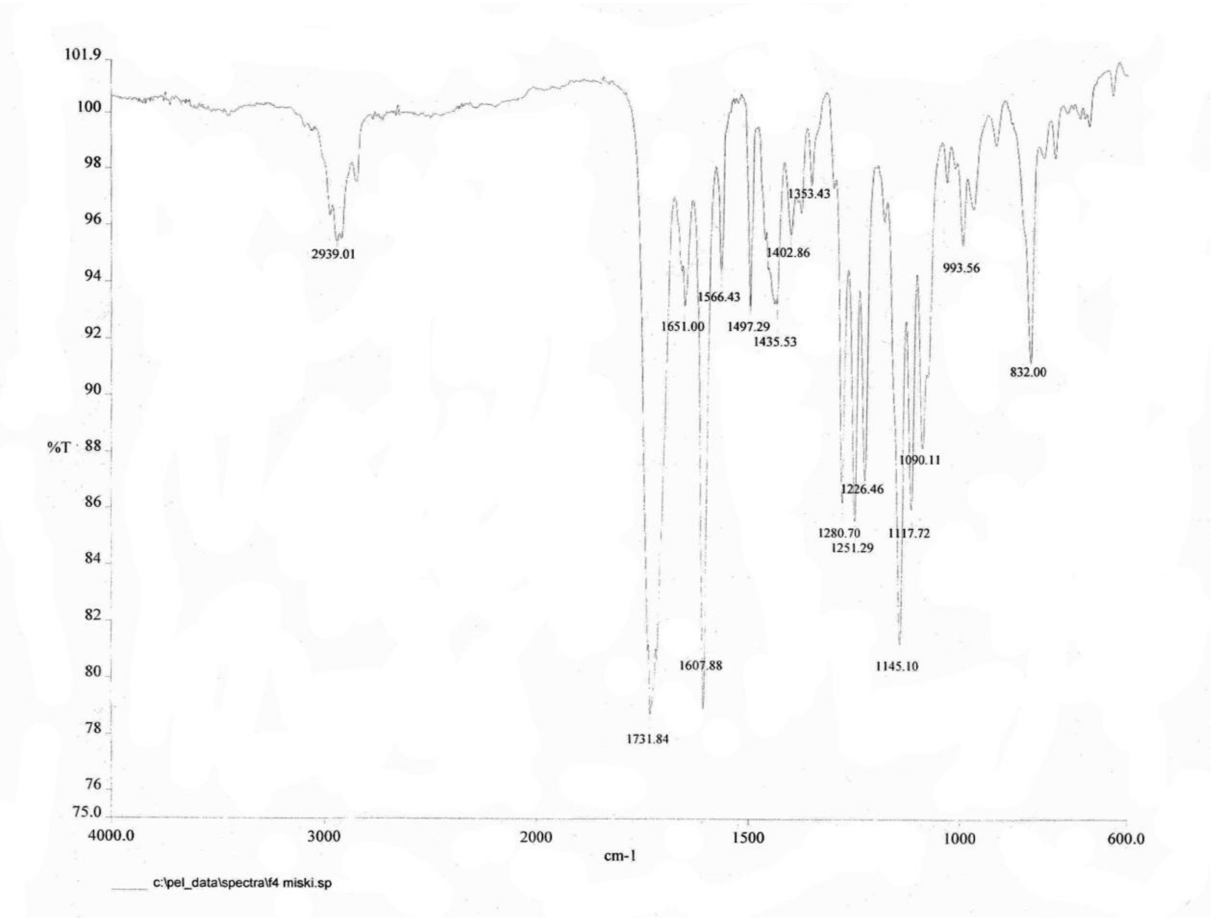

Figure S2. IR spectrum (in KBr) of 4'-Senecioiloxyosthol (**1**).

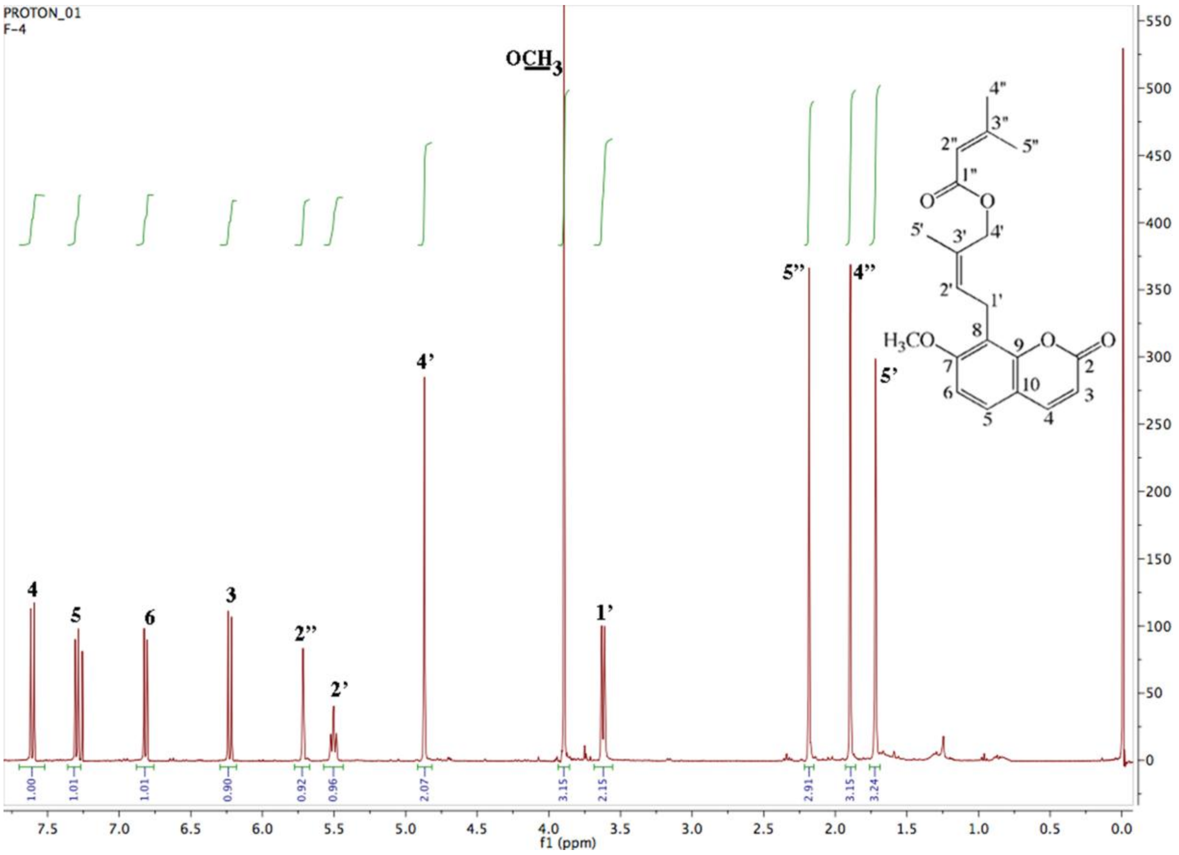

Figure S3. ^{1}H-NMR spectrum (400 MHz, in $CDCl_3$) of 4’-Senecioiloxyosthol (**1**).

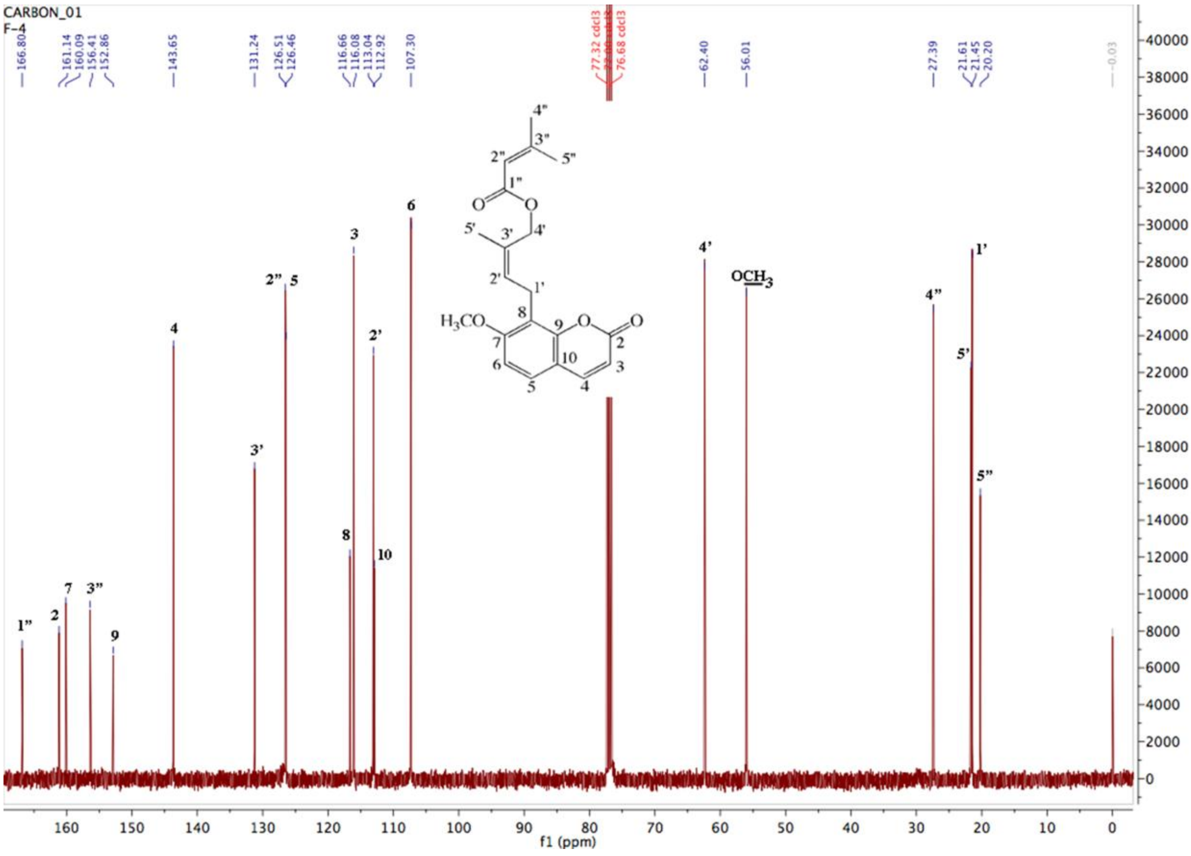

Figure S4. ^{13}C-NMR spectrum (100 MHz, in CDCl3) of 4’-Senecioiloxyosthol (**1**).

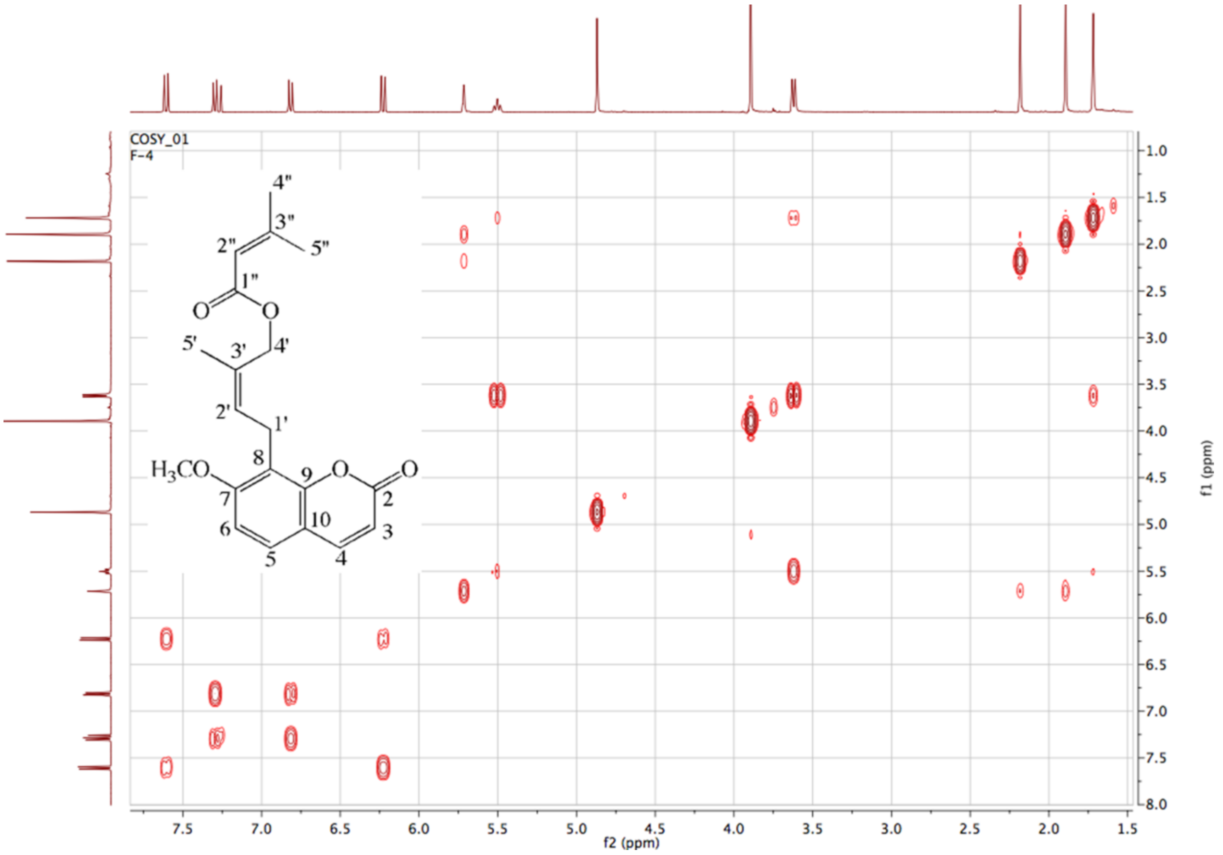

Figure S5. ^{1}H -^{1}H COSY spectrum of 4’-Senecioiloxyosthol (**1**).

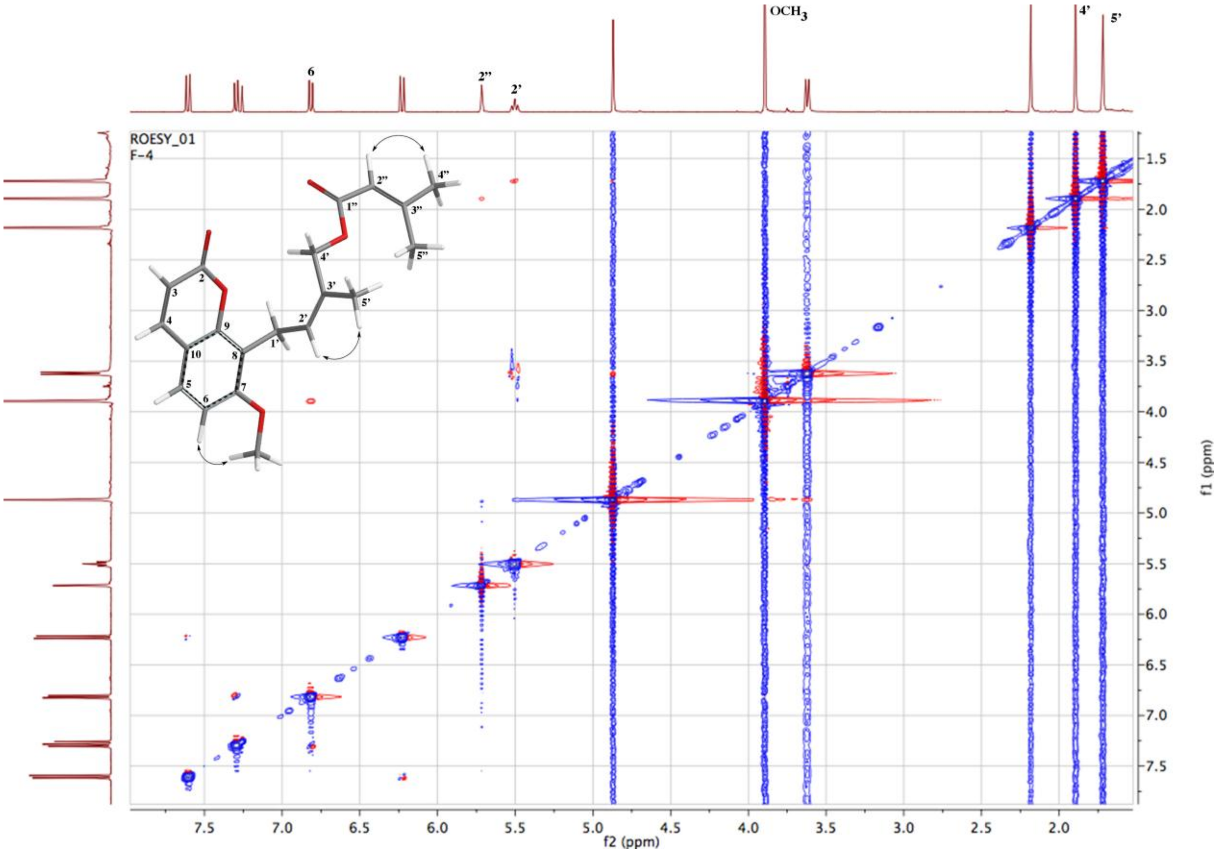

Figure S6. ROESY spectrum of 4’-Senecioiloxyosthol (**1**).

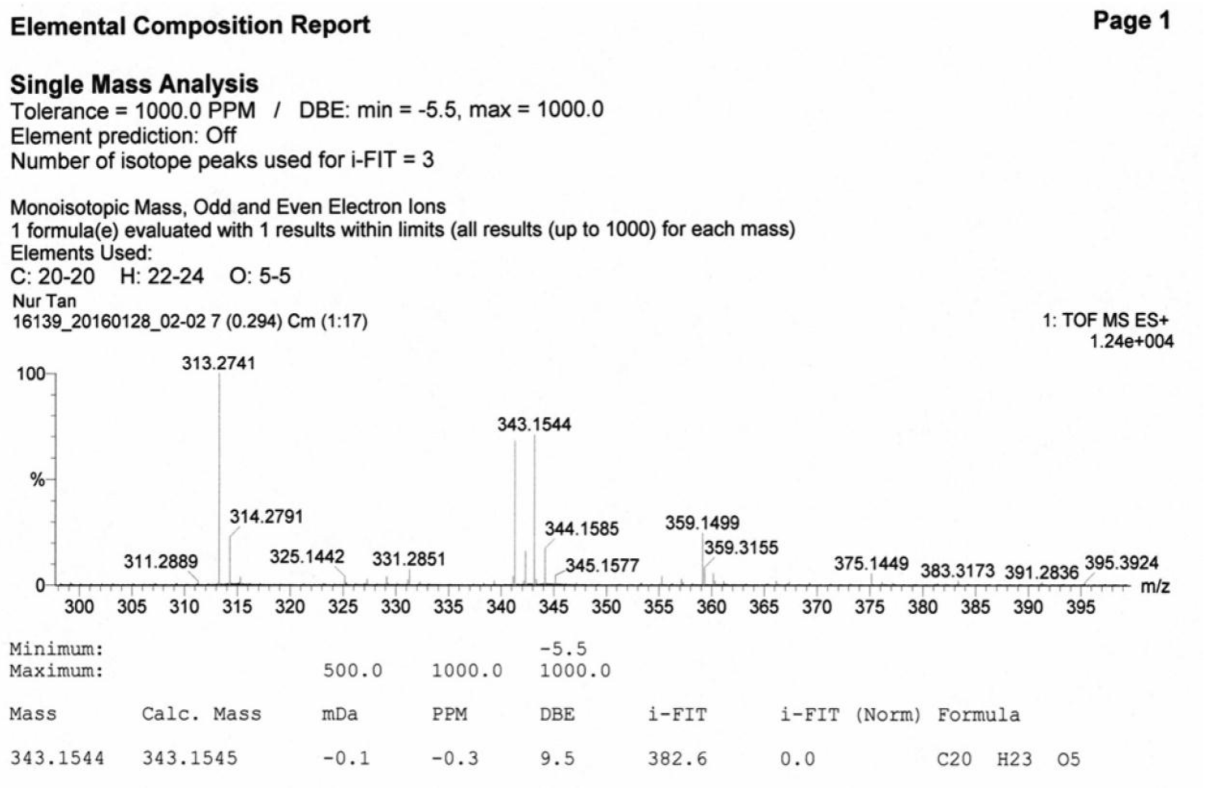
Elemental Composition Report Page 1

Single Mass Analysis
Tolerance = 1000.0 PPM / DBE: min = -5.5, max = 1000.0
Element prediction: Off
Number of isotope peaks used for i-FIT = 3

Monoisotopic Mass, Odd and Even Electron Ions
1 formula(e) evaluated with 1 results within limits (all results (up to 1000) for each mass)
Elements Used:
C: 20-20 H: 22-24 O: 5-5
Nur Tan
16139_20160128_02-02 7 (0.294) Cm (1:17) 1: TOF MS ES+ 1.24e+004

Minimum:				-5.5			
Maximum:		500.0	1000.0	1000.0			
Mass	Calc. Mass	mDa	PPM	DBE	i-FIT	i-FIT (Norm)	Formula
343.1544	343.1545	-0.1	-0.3	9.5	382.6	0.0	C20 H23 O5

Figure S7. HRESIMS spectrum of 4'-Senecioiloxyosthol (**1**).

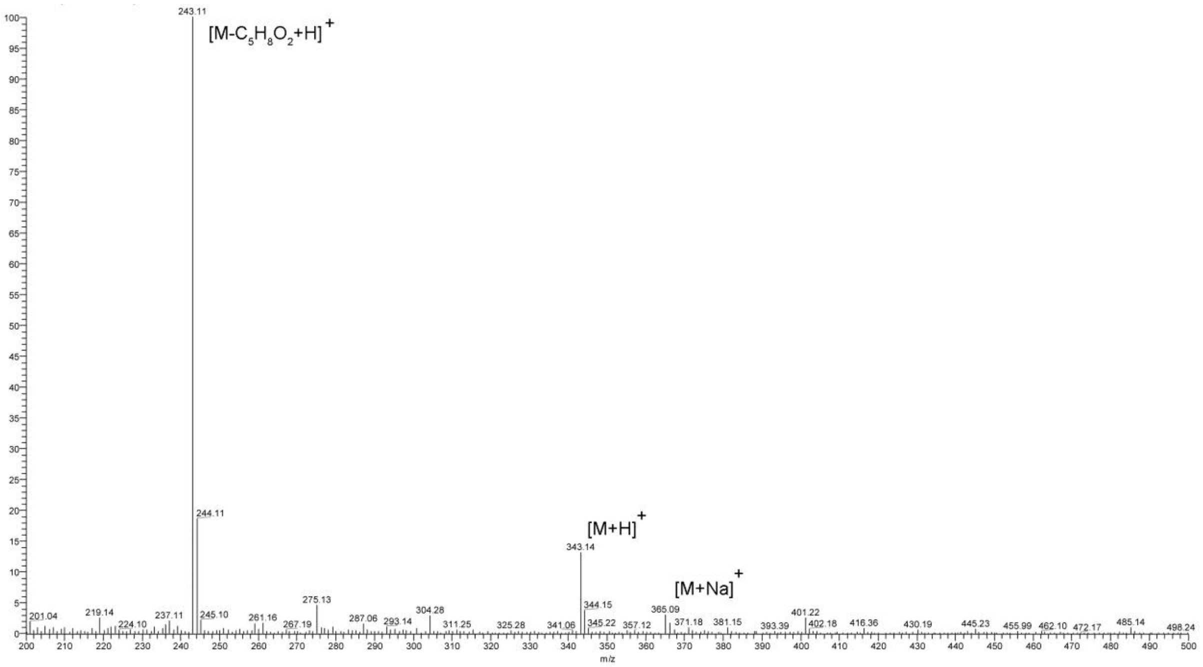

Figure S8. ESIMS spectrum of 4'-Senecioiloxyosthol (**1**).
